# Predictive modeling of sensory responses in deep brain stimulation

**DOI:** 10.3389/fneur.2024.1467307

**Published:** 2024-10-01

**Authors:** László Halász, Bastian E. A. Sajonz, Gabriella Miklós, Gijs van Elswijk, Saman Hagh Gooie, Bálint Várkuti, Gertrúd Tamás, Volker A. Coenen, Loránd Erōss

**Affiliations:** ^1^Institute of Neurosurgery and Neurointervention, Faculty of Medicine, Semmelweis University, Budapest, Hungary; ^2^Albert Szent-Györgyi Medical School, Doctoral School of Clinical Medicine, Clinical and Experimental Research for Reconstructive and Organ-Sparing Surgery, University of Szeged, Szeged, Hungary; ^3^Department of Stereotactic and Functional Neurosurgery, Medical Center of Freiburg University and Medical Faculty of Freiburg University, Freiburg, Germany; ^4^János Szentágothai Doctoral School of Neurosciences, Semmelweis University, Budapest, Hungary; ^5^CereGate GmbH, München, Germany; ^6^Department of Neurology, Faculty of Medicine, Semmelweis University, Budapest, Hungary; ^7^Center for Deep Brain Stimulation, Freiburg University, Freiburg, Germany

**Keywords:** DBS programming, paresthesia, machine learning, computer-brain interfaces, prediction

## Abstract

**Introduction:**

Although stimulation-induced sensations are typically considered undesirable side effects in clinical DBS therapy, there are emerging scenarios, such as computer-brain interface applications, where these sensations may be intentionally created. The selection of stimulation parameters, whether to avoid or induce sensations, is a challenging task due to the vast parameter space involved. This study aims to streamline DBS parameter selection by employing a machine learning model to predict the occurrence and somatic location of paresthesias in response to thalamic DBS.

**Methods:**

We used a dataset comprising 3,359 paresthetic sensations collected from 18 thalamic DBS leads from 10 individuals in two clinical centers. For each stimulation, we modeled the Volume of Tissue Activation (VTA). We then used the stimulation parameters and the VTA information to train a machine learning model to predict the occurrence of sensations and their corresponding somatic areas.

**Results:**

Our results show fair to substantial agreement with ground truth in predicting the presence and somatic location of DBS-evoked paresthesias, with Kappa values ranging from 0.31 to 0.72. We observed comparable performance in predicting the presence of paresthesias for both seen and unseen cases (Kappa 0.72 vs. 0.60). However, Kappa agreement for predicting specific somatic locations was significantly lower for unseen cases (0.53 vs. 0.31).

**Conclusion:**

The results suggest that machine learning can potentially be used to optimize DBS parameter selection, leading to faster and more efficient postoperative management. Outcome predictions may be used to guide clinical DBS programming or tuning of DBS based computer-brain interfaces.

## Introduction

Deep brain stimulation (DBS) has emerged as a promising treatment for a variety of neuropsychiatric diseases (1). It is established for movement disorders such as Parkinson’s disease (2–4), essential tremor (5, 6), and dystonia (7, 8), and for epilepsy (9, 10). It is an emerging therapy in neuropathic pain (11) and selected psychiatric disorders like major depression and obsessive-compulsive disorder (12, 13). Despite its clinical efficacy, DBS is frequently accompanied by undesirable side effects, which can adversely affect patient satisfaction and treatment outcomes (14–16). Conversely, in emerging technologies such as computer-brain interfaces (CBI), implanted electrodes can be used to deliberately induce sensations like paresthesias (17, 18).

With respect to clinical applications, major challenges in the post-implant management of DBS therapy remain. In particular, optimizing stimulation parameters presents a significant bottleneck in DBS therapy. One of the most important factors is searching the vast parameter space involved in DBS programming, which is often done manually (15, 19). Computer algorithms offer significant potential to improve the efficiency and accuracy of DBS parameter selection, whether the intended outcome is maximizing symptom reduction, minimizing side effects, or eliciting specific sensations for CBI applications. This work addresses the feasibility of using machine learning to improve the efficiency of DBS parameter selection, potentially overcoming some of these limitations.

Understanding the factors that contribute to paresthesias and devising strategies to mitigate side effects are essential to optimize DBS therapy. It is proposed that paresthesias result from the activation of sensory afferents near the DBS lead (20–22). Consequently, in DBS for tremor, for example, paresthesia induction can signal the activation of critical anatomical landmarks, such as the medial lemniscus and the posterior-medial border of the cerebellothalamic tract (21, 22).

One commonly used method to assess the effects of DBS is to simulate the Volume of Tissue Activation (VTA), which estimates the spatial extent of neural activation in response to stimulation (23–25). VTA models primarily rely on stimulation pulse width and current amplitude and may not fully capture the intricate relationships among all relevant stimulation parameters, individual neuroanatomy, tissue heterogeneity, and their effects on perceptual phenomena (26–28).

Here we aimed to predict the occurrence and spatial localization of paresthesias in response to thalamic DBS. To achieve this, we used a dataset comprising more than three thousand paresthesia location records, empirically obtained from 18 thalamic DBS leads in 10 individuals across two clinical centers. We modeled the VTA for each stimulation trial. Stimulation parameters and VTA metrics were used as input variables for the prediction models, which were evaluated using cross-validation.

## Methods

### Study design

This is a cross sectional study of a single cohort of 10 individuals (three females), from two independent deep brain stimulation centers. We recruited people who had chosen deep brain stimulation (DBS) surgery to treat either tremor or chronic neuropathic pain. Demographics and the anatomical target regions of the DBS leads (model DB2202, Boston Scientific Corporation, Marlborough, MA, United States) are summarized in Table 1. The implantations took place between July 2019 and October 2020. The experiments took place between 1 and 4 days after the surgery, during the period when externalized extension cables were connected to the DBS leads. We conducted the experiments in accordance with local guidelines and regulations and in accordance with the Declaration of Helsinki. The ethics committees of both centers approved the study (agreement number 235/19 for Freiburg; OGYEI/23818/2019 for Budapest). All participants provided written informed consent prior to the experimental procedures.

**Table 1:** Patient demography and implantation information.

Patient	Sex	Age	Indication	DBS lead locations	Center
p1	M	72	Pain	R-VPL/VPM, R-PVG/PAG*	Freiburg
p2	M	72	Pain	R-VPL/VPM, R-PVG/PAG*	Freiburg
p3	M	60	Essential Tremor	R-VIM, L-VIM	Freiburg
p4	M	70	Essential Tremor	R-VIM, L-VIM	Freiburg
p5	M	66	Essential Tremor	R-VIM, L-VIM	Freiburg
p6	M	74	Essential Tremor	R-VIM, L-VIM	Budapest
p7	M	71	Parkinson’s disease	R-VIM, L-VIM	Budapest
p8	F	79	Essential Tremor	R-VIM, L-VIM	Budapest
p9	F	74	Essential Tremor	R-VIM, L-VIM	Budapest
p10	F	72	Essential Tremor	R-VIM, L-VIM	Budapest

PVG/PAG: periventricular and periaqueductal gray. VPL/VPM, ventroposterolateral and ventroposteromedial thalamic nuclei. VIM, ventral intermediate thalamic nucleus. Leads indicated with * were not used in this study.

### Brain imaging

#### Magnetic resonance imaging

Magnetic resonance imaging (MRI) was performed without a stereotactic frame. MR examinations comprised T1-weighted, T2-weighted sequences.

In Freiburg, MR imaging was performed 1–3 days before surgery, and - if necessary - with the person under mild sedation. MR images were acquired on a whole-body 3 T MR system (PRISMA, Siemens Healthcare, Erlangen, Germany) using a 64 channel phased array head coil. For T1-weighted imaging a magnetization-prepared rapid gradient-echo (MP-RAGE) scan was used (TR 2,300 ms, TE 2.26 ms, flip angle 12^°^, FOV 256 mm, voxel size 1mm^3^). The T2-weighted scan was a fast spin-echo sequence (TR 2,500 ms, TE 231 ms, FOV 256 mm, voxel size 1mm^3^).

In Budapest, MR imaging was performed between 1 month and 5 days before surgery, under general anesthesia. MR images were acquired on a 3 T whole-body magnetic resonance imaging system (Philips Healthcare, Best, The Netherlands) using an 8-element phased array head coil. For T1-weighted imaging a magnetization-prepared rapid gradient-echo (MP-RAGE) scan was used (TR 8.5 ms, TE 3.8 ms, flip angle 8^°^, FOV 256 mm, reconstructed to 1mm^3^ voxels). The T2-weighted scan was a fast spin-echo sequence (TR 12.65 ms, TE 100 ms, FOV 254 mm, reconstructed to 1.44mm^3^ voxels).

#### Computed tomography

Pre-operative and post-operative computed tomography scans (CT) were both performed on the day of surgery, with the exception of p10 whose post-operative CT was taken the day after surgery. Pre-operative CT was acquired to register the stereotactic frame to the person’s anatomy. Post-operative CT was acquired to determine the positions of the implanted DBS electrode arrays.

In Freiburg, CT scans were acquired with a SOMATOM Definition AS scanner (Siemens Healthcare, Erlangen, Germany). The parameters of the preoperative CT were as follows: tube voltage 120 kV, tube current 365 mAs, collimation 19·0.6 mm, tube rotation time 1.0 s, pitch 0.55, matrix 512·512, section thickness 1.5 mm, increment 1.5 mm. The parameters of the post-operative CT were identical, except for a tube current of 320 mAs. The different post-operative scanning parameters were chosen for better electrode metal artifact suppression.

In Budapest, CT scans were acquired with a Brilliance 8,000 16-row multidetector scanner (Philips Healthcare, Best, Netherlands). The parameters of the preoperative CT were as follows: tube voltage 120 kV, tube current 350 mAs, collimation 16·0.75 mm, tube rotation time 1.0 s, pitch 0.942, matrix 512·512, section thickness 1.5 mm, increment 1.5 mm. The parameters of the post-operative CT were identical, except for the tube rotation time of 0.75 s, pitch of 0.688, section thickness of 2 mm, and an increment of 1 mm. The different post-operative scanning parameters were chosen for better electrode metal artifact suppression.

### Brain stimulation

We performed the experiments in dedicated laboratory rooms in each of the two clinics. The participant sat on a chair, behind a regular office desk. At a distance of about 60–80 cm from the participant, there was a PC monitor to provide feedback to the participant during the experiment. The two participants with two leads implanted on the same were only stimulated through their thalamic electrodes. We delivered the stimuli with an external programmable neurostimulator. In Freiburg, we used a Neuro Omega stimulator (Alpha Omega Engineering, Nof HaGalil, Israel). In Budapest we used a CereStim stimulator (BlackRock, Salt Lake City, UT, USA). We connected the neurostimulator and the DBS lead using a surgical operating room cable (SC-4108; Boston Scientific, Marlborough, MA, USA). We used custom software written in Matlab (MathWorks, Natick, MA) to send instructions to the external neurostimulator interface using the manufacturer’s software development kit. We asked participants to provide feedback about stimulation-induced sensations, either orally or via a button box (ResponsePixx; VPixx Technologies, Saint-Bruno, QC, Canada), depending on their preference. The range of stimulation parameters was restricted to the established safety ranges for deep brain stimulation. Stimulation parameters included the following ranges: pulse frequency 20–205 Hz, pulse width 50–250 μs, and pulse amplitude 0.25–3.0 mA. With the CereStim stimulator, electrode contact number 8 was used for the return current path. With Neuro Omega, contact 8 was used as a passive ground. To keep the applied waveforms comparable between the different stimulators, we generated a basic waveform that mimics monophasic passive discharge. This waveform was the default throughout the experiments.

### Data analysis

#### Image processing

Image processing was performed on a “NeuroImaging Tools and Resources Collaboratory Cloud Computing Environment” (NITRC-CE) (29), running on an Amazon Web Services EC2 P3 virtual machine equipped with NVIDIA Tesla V100 GPUs. DICOM images were converted to compressed NIFTI format using MRICron’s dcm2niix converter (30). Tools available in the FMRIB Software Library (FSL 6.0.6.2) were used for linear and non-linear image registrations.

#### Modeling of volume of tissue activated

The Volume of Tissue Activated (VTA) was modeled using Lead-DBS 2.6 (31) software with the FastField (32) modeling method, running in Matlab (version 2023a). For each stimulation trial, we used the respective electrode configuration, stimulation currents, and cathodic pulse width as input parameters for the VTA calculation. The axon diameter parameter was fixed to 3.5 μm. DBS contact positions and orientation were determined from the post-operative CT images using the DiODe algorithm from Lead-DBS, and subsequently visually verified using Brainlab Elements (Brainlab AG, Munich, Germany). To allow pooling of cases, the DBS contact positions were transformed from individual space into MNI space. The transformation matrix was determined by non-linear registration (12 degrees of freedom) of the person’s T1 MRI to the 0.5mm^3^ MNI ICBM 2009b NLIN ASYM brain template (MNI hereafter) using ANTs (33). Subsequently, the VTA modeling was done in MNI space. For further analysis, VTAs were converted into binary masks.

#### Data augmentation and preparation

During the experiment, in the majority of trials stimulation was applied above the sensation threshold. Thus, the empirical dataset contained relatively few instances in which no paresthesias were reported. However, our aim extended beyond predicting the somatic area of evoked sensations; we also aimed to predict the presence or absence of sensations altogether. To address this, we augmented the empirical dataset by introducing pseudotrials that were synthesized from existing empirical data. Pseudotrials were generated from the subset of stimulation trials where no paresthesias were reported. This synthesis involved systematically generating novel combinations of pulse width, frequency, and stimulation currents below the empirically observed sensation thresholds.

The empirical dataset included multiple identical stimulation and response instances (e.g., due to repeating stimulation with identical parameters). We retained only those trials with distinct combinations of stimulation parameters and responses. This also ensured that during cross-validation, the test splits exclusively comprised data patterns that had not been encountered at all during the training phase. Subsequently, a random subset (sampled without replacement) of the pseudotrials were added to the set of distinct empirical trials to balance out the dataset such that for each individual DBS lead equal proportions of trials with and without sensations were available. The augmented dataset, comprising both empirical trials and synthetically generated pseudotrials, was used for further processing, including VTA generation.

Spatial data that are related to right-sided DBS electrodes were flipped so that all data is virtually projected onto the left hemisphere (34, 35). Consequently, analyses were performed in a unilateral frame of reference. For reported paresthesias, contralateral denotes the hemibody opposite to the stimulation location.

#### Definition and cross-validation of prediction models

For the predictions, a series of binary classifiers were trained. One binary classifier was trained to predict whether any sensation would occur or not (i.e., without predicting in which specific body parts). For this classifier we only provided stimulation-energy related parameters as features (i.e., *Pw*, *Freq*, *Current*, *Hemisphere*; see Table 2). Since the purpose of this classifier is to predict whether or not any sensation occurs (without concern for the specific location in the body), we limited the features to energy-related parameters. These features are expected to correlate with whether a sensation is triggered but should not reveal where the sensation occurs. Preserving spatial information for the specific somatic predictions also avoided redundancy. To predict the occurrence of sensations in specific body parts, a separate binary classifier was trained for each somatic area (e.g., finger, wrist, etc.). To these somatic prediction models we provided additional features about the spatial location of the stimulation energy (i.e., *VTAspread*, *VTAx*, *VTAy*, *VTAz*, *Freq*, *Hemisphere*; see Table 2).

**Table 2:** Features used for the prediction models and their definitions.

Abbrevation	Definition	Paresthesia	Somatic	Control
Pw	Pulse width: duration of each cathodic stimulation pulse delivered	*	*	*
Freq	Frequency: number of cathodic stimulation pulses delivered per second	*	*	*
Current	Total cathodic current delivered each stimulation pulse	*	*	*
Hemisphere	Brain hemisphere where stimulation was applied	*	*	*
VTAx	X coordinate in MNI space of the VTA centroid		*	
VTAy	Y coordinate in MNI space of the VTA centroid		*	
VTAz	Z coordinate in MNI space of the VTA centroid		*	
VTAspread	Spatial spread of the VTA, mean distance of voxels to centroid		*	

**Figure 1:**
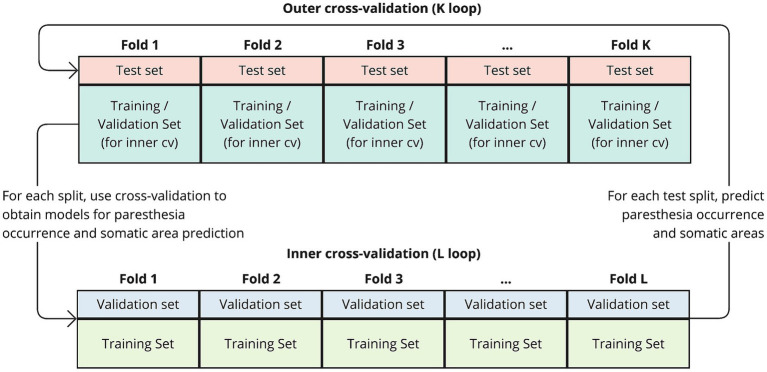
Schematic of the nested cross-validation procedure. The outer K loop iterates over the data, splitting it into training-and-validation and test sets. In each iteration, a portion of the data is held out as a test set (outer fold), while the remaining data is used for training and validation. Within each K fold, the inner L loop further splits the training set into multiple folds to perform cross-validation. The prediction models are trained on the training folds from the inner loop and evaluated on the validation folds. After completing the inner loop, resulting models are then evaluated using the test set from the outer K fold.

The prediction models used the LogitBoost algorithm for classification and were trained and evaluated using a nested two-level (K by L) cross-validation approach (illustrated in Figure 1). In this procedure, the outer K loop iterates over the data, splitting it into training-and-validation and test sets. We used two different approaches for outer K-fold splitting, which is further explained in the next section “Evaluation of predictions.” The inner L loop further splits the K-th training set into multiple folds. Prediction models were trained using cross-validation on the inner L folds. The splits in the inner loop were based on group folding by lead-id, ensuring that all trials from the same lead were held out for validation, thus enhancing generalization. Within each L iteration, the training-validation subset for each somatic prediction model was rebalanced so that both classes (True/False) had an equal number of occurrences. This was achieved by identifying the least frequent class and randomly dropping trials from the more frequent class. Trials where no sensations occurred, and thus no somatic areas were reported, were excluded in the training of the somatic prediction models. Only somatic prediction models with a Kappa value above 0.05 after L-fold cross-validation were retained. These models were then evaluated using the test sets from the outer K fold.

Model training and evaluation analyses were carried out in R (version 4.4).

#### Evaluation of predictions

We evaluated the predictions in two scenarios, which differed in the way the training-and-validation and test splits were created:

- Intra-sample evaluation: The K test sets consisted of a random sample of trials with parameter-response combinations distinct from all trials in the training and validation sets. However, these hold-out trials were drawn from cases (i.e., leads) that were also included in the L-loop training and validation cycles. This means the test set includes trial instances the model has not explicitly been trained on, but these still come from the set of cases that were part of the training/validation process. Intra-sample performance reflects the model’s ability to generalize and predict outcomes for new stimulation parameter combinations within the same individuals used for training (i.e., like patients for whom prior responses are on record).
- Inter-sample (leave-cases-out) evaluation: The K test sets only included trials from cases (i.e., leads) that were entirely excluded from the L-loop iterations of the cross-validation process. This means the test set contains data from cases that were completely unseen during the L-loop training and validation. These trials represent truly unseen data from new cases (i.e., similar to *de novo* patients), providing a more challenging evaluation of the model’s generalization ability.

In both scenarios, resampling with replacement was used to ensure a uniform number of trials per case in the test set. The number of trials per case was set to the average trial count across cases. This approach ensured that each case contributed equally to the aggregated test performance metrics, while maintaining the original overall size of the test set. The model’s performance was evaluated on two aspects: (1) Prediction of the occurrence of paresthesia (“paresthesia predictions”), and (2) prediction of the somatic locations where a paresthesia would occur (“somatic predictions”). Since stimulation could elicit paresthesias in none, one, or multiple body regions simultaneously, predicting the somatic locations is a multi-output task, where multiple target variables are predicted in parallel. Thus, for each trial, the paresthesia prediction and the multiple somatic predictions were read out in parallel and combined. The somatic predictions in a given trial were retained only if the paresthesia prediction was positive. Otherwise, all somatic area predictions were overridden to *False*.

We used Kappa as the primary metric to compare model performance. Kappa is a robust measure of agreement that accounts for chance. In addition, we used accuracy, sensitivity, specificity, precision, F1-score. For the somatic prediction metrics the prediction of each of the multiple binary target variables was taken into account and aggregated. Our “distance” metric summarizes the average difference between observed and predicted composite somatic response (“thumb + finger + wrist”). A distance of 0 indicates an exact agreement between predictions and observations, whereas a distance of 0.5 signifies chance-level agreement, implying predictions are no better than random guessing. We report descriptive statistics as averages followed by the associated standard deviation, unless stated otherwise. The model performance metrics were aggregated across the K iterations. For statistical comparison of model metrics between scenarios, we used the non-parametric Wilcoxon rank sum test.

#### Control analyses

To evaluate the contribution of VTA information to somatic predictions, we conducted a control analysis. In this analysis, we repeated the nested cross-validation and evaluation procedure described earlier, but excluded the spatial features. Instead, we provided the model with *Pw, Freq, Current, Hemisphere*—the same features used for the paresthesia occurrence prediction model.

Additionally, we assessed the performance of two types of naive prediction models and used their metrics as baselines for comparison against actual predictions. The first naive model generated predictions by randomly permuting the reference (ground truth) response data. Consequently, its performance was influenced by the underlying class distribution. The second naive model always predicted True for both paresthesia occurrence and somatic area prediction.

## Results

**Figure 2:**
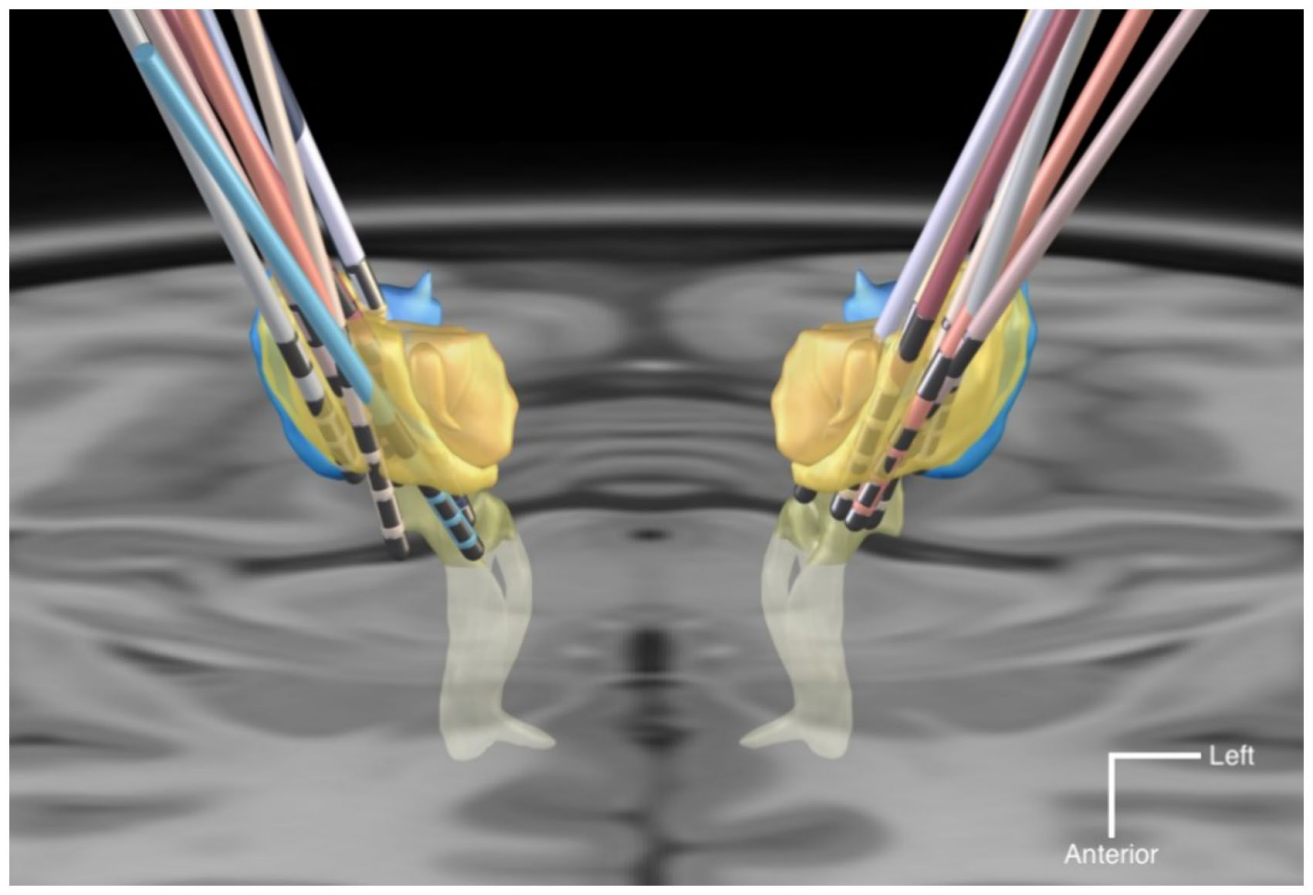
Graphic rendering of the 18 DBS leads with anatomical landmark structures, in a common space. The anatomical structures are sourced from the DISTAL atlas (54). VIM is colored in yellow; VPL/VPM in blue; Medial Lemniscus in light-yellow. The background is an axial T1-MRI slice (z = 0 mm) of the MNI template. The mean coordinates (±SD) of the deepest contact points of the VIM-targeted leads were *x* = −12.6 (±2.1), *y* = −19.8 (±1.8), *z* = −3.9 (±1.8) in the left hemispheres, and *x* = 13.6 (±1.6), *y* = −19.6 (±1.7), *z* = −3.5 (±1.9) in the right hemispheres. For the VPL-targeted leads these were *x* = 10.1 (±0.4), *y* = −24.2 (±1.7), *z* = −7.8 (±0.9).

**Figure 3:**
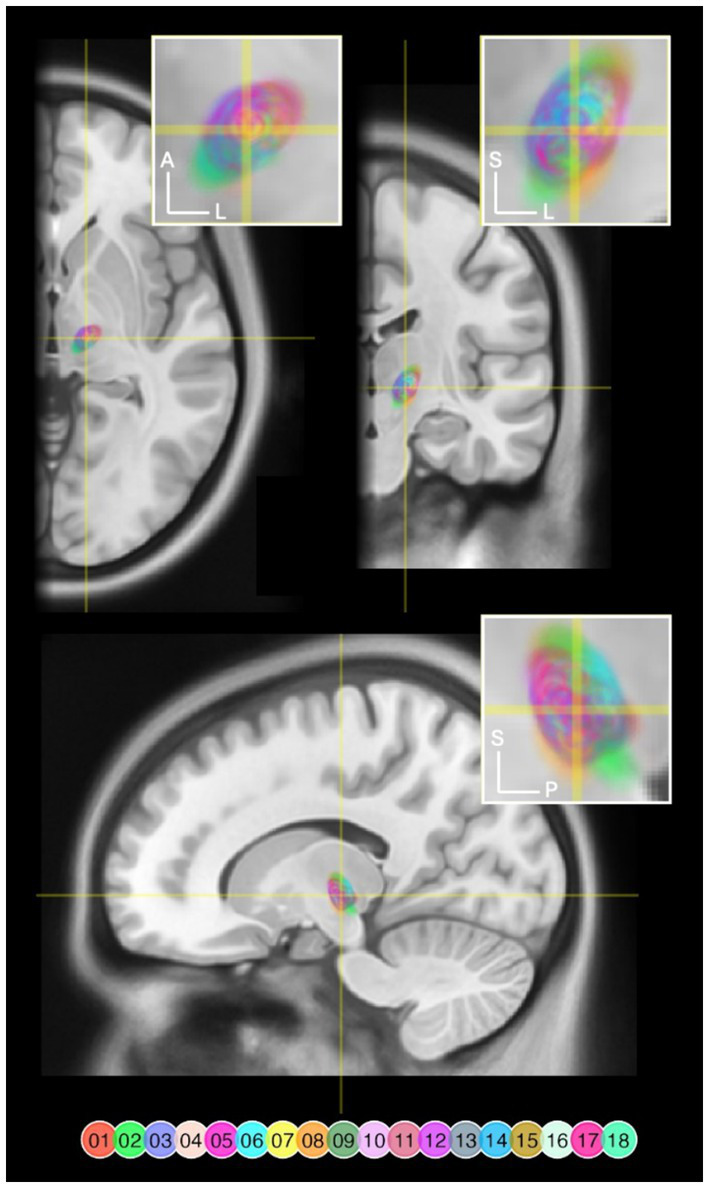
VTAs rendered and overlaid on a MNI T1 Magnetic Resonance Image (MRI) in axial (*z* = 141), coronal (*y* = 232), and sagittal (*x* = 170) slices. Right-sided VTAs were mirrored so that these are projected onto the left hemisphere. The crosshairs indicate where the slices intersect. The yellow rectangles delineate the target areas of each magnification inset. VTA pixels are color coded by lead number and flattened along the axis that is not visible in that specific view. VTA pixels are transparent to allow overlapping areas to blend. A, anterior; L, left; S, superior; P, posterior.

A three-dimensional overview of all 18 DBS leads, rendered together with anatomical landmarks in a common space is provided in Figure 2. A triple-plane orthographic rendering of all the VTAs in the dataset is shown in Figure 3.

**Figure 4:**
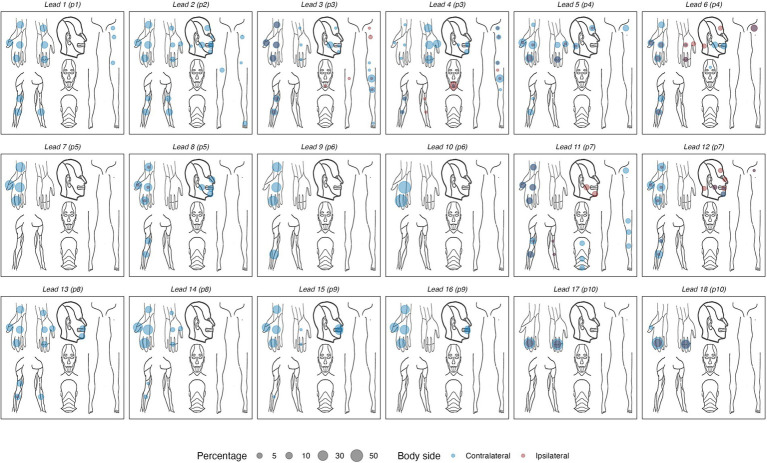
Body maps showing the locations of reported paresthesias for each of the 18 DBS electrode arrays. Left- and right-sided sensations are collapsed into a unilateral frame of reference, using a color code to indicate contra- vs. ipsilateral sensations. Percentages reflect the proportion of all reported sensations in each body area, calculated per DBS lead.

**Figure 5:**
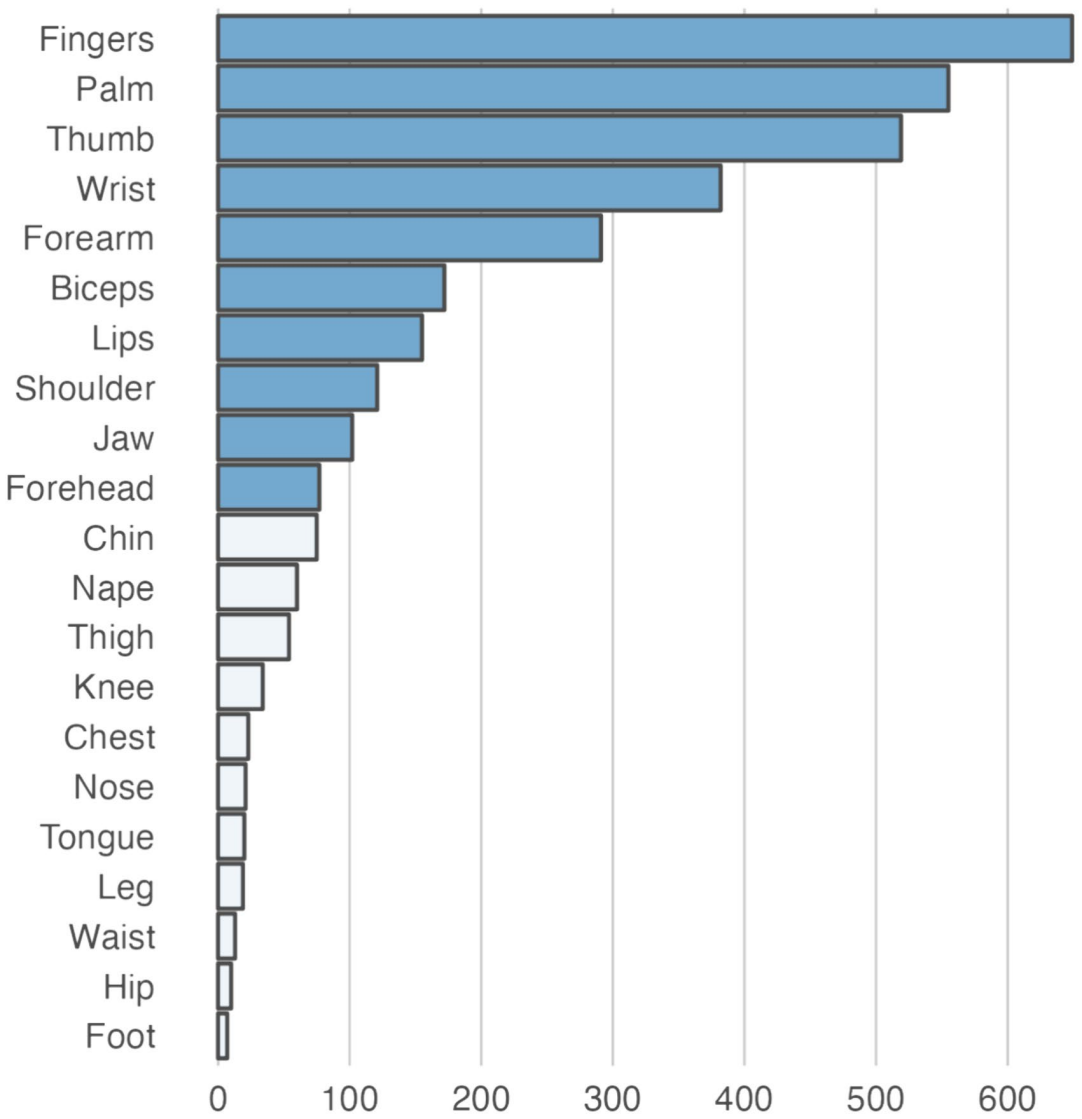
Total number of paresthesias recorded for each somatic area in the dataset used for training and testing the prediction models. The darker shaded bars represent somatic areas for which specific prediction models were trained and tested. For the remaining somatic areas (light shaded bars), no direct prediction models were trained. However, these areas were used as negative instances in other somatic area models, representing trials where the target sensation of the respective model did not occur (e.g., chin paresthesias could act as False instances for a model predicting paresthesias in the fingers).

The empirical data from the experiment that were used as the basis for the training and evaluation are summarized in Table 3. Across the empirical dataset, the ratio between trials with paresthetic sensations vs. without was 87 vs. 13%. Figure 4 shows an overview of the somatic areas in which paresthesias were reported. Each lead evoked paresthesias in at least 2 different somatic areas, with the exception of lead 17 (participant 10) for which paresthesias were evoked exclusively in the fingers. On average (±SD), a DBS lead evoked paresthesias in 8.4 ± 5.1 different somatic areas. Paresthesias were most frequently reported in the fingers, palm (of the hand), and the thumb. For training and evaluation of the prediction model we retained the subset of empirical trials with distinct combinations of feature and response values. In total, 667 pseudotrials were added to balance trials with and without paresthesias. This resulted in a total of 1,451 unique trials, with a 50 − 50% balance between no-paresthesia and paresthesia trials. In total, the prediction dataset comprised 3,359 reported paresthesias across 21 different body regions. The somatic areas from the trials in the prediction dataset, and their counts, are summarized in Figure 5 and in Supplementary Figure S1.

**Table 3:** Counts and percentages of stimulation trials and their responses obtained in the experiment.

Lead id	Patient id	Paresthesias	No paresthesia
1	p1	67 (97.1%)	2 (2.9%)
2	p2	147 (95.5%)	7 (4.5%)
3	p3	96 (90.6%)	10 (9.4%)
4	p3	82 (95.3%)	4 (4.7%)
5	p4	347 (89.7%)	40 (10.3%)
6	p4	226 (91.5%)	21 (8.5%)
7	p5	47 (90.4%)	5 (9.6%)
8	p5	197 (92.9%)	15 (7.1%)
9	p6	20 (95.2%)	1 (4.8%)
10	p6	17 (100.0%)	0 (0.0%)
11	p7	225 (79.5%)	58 (20.5%)
12	p7	174 (87.9%)	24 (12.1%)
13	p8	107 (69.5%)	47 (30.5%)
14	p8	84 (70.6%)	35 (29.4%)
15	p9	131 (86.8%)	20 (13.2%)
16	p9	13 (72.2%)	5 (27.8%)
17	p10	124 (85.5%)	21 (14.5%)
18	p10	95 (88.0%)	13 (12.0%)

The paresthesias column gives the number (and percentage) of trials in which paresthesias were reported in one or multiple somatic regions.

The allocation of cases across K and L splits in the inter-sample (leave-case-out) cross-validation scenario is shown in Supplementary Figure S2. There were 8 K splits, each containing 5 L splits. The fold creation resulted in an average split ratio of 87.5% (training) vs. 12.5% (testing) for the K-folds, and 80% (training) vs. 20% (validation) within the L-folds.

**Figure 6:**
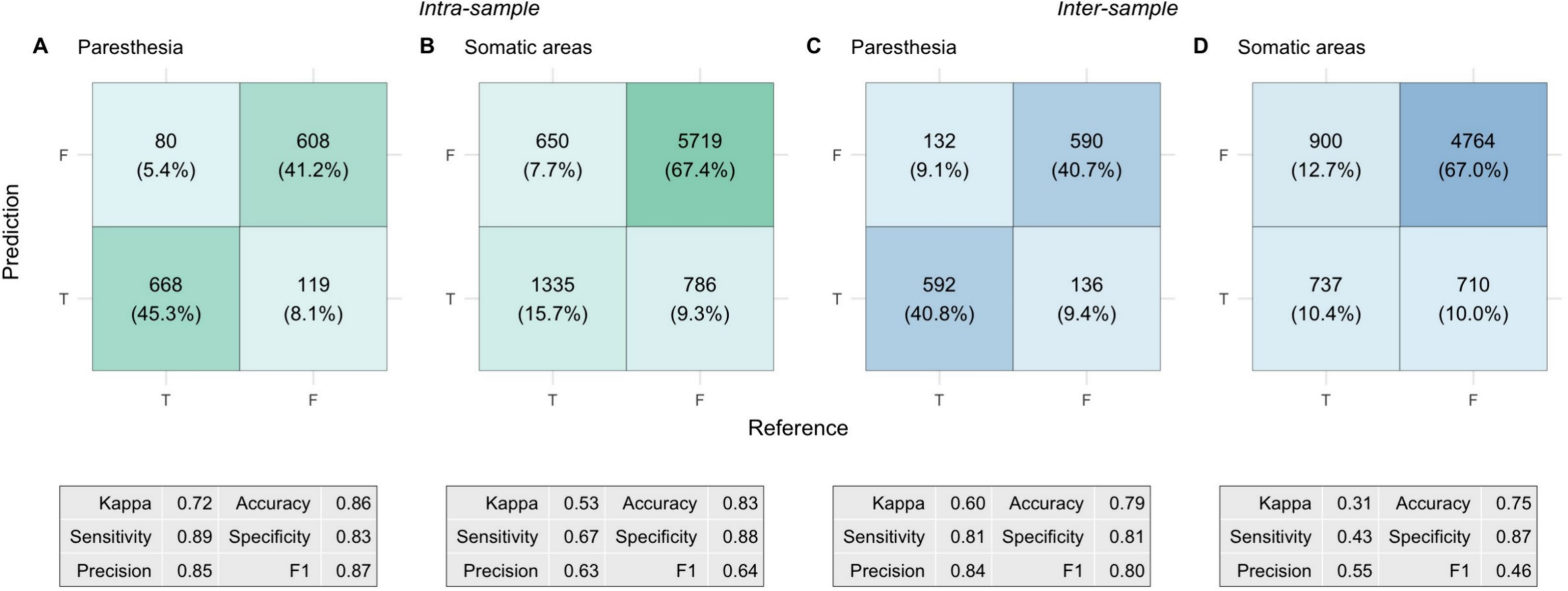
Confusion matrices and performance metrics from the nested cross-validation of prediction models. Each cell shows the total count of true/false positives/negatives, with the corresponding percentage in parentheses. Percentages represent the proportion of total predictions classified as True (paresthesia occurring) or False (paresthesia not occurring), with model predictions on the vertical axis and the reference (ground truth) on the horizontal axis. The panels depict: **(A)** Intra-sample paresthesia predictions, **(B)** Intra-sample somatic predictions, **(C)** Inter-sample paresthesia predictions, and **(D)** Inter-sample somatic predictions. The tables beneath each matrix show the corresponding performance metrics.

The predictions and associated performance metrics of the model in the two different scenarios are summarized in Figure 6. Paresthesia prediction performance was comparable between the intra-sample and the inter-sample scenarios. Although the performance metrics suggested worse performance in the latter scenario, the difference in Kappa was not significant (0.72 ± 0.11 vs. 0.60 ± 0.26; *p* = 0.22).

For the somatic predictions, the difference between the two scenarios was more pronounced. Kappa was significantly lower in the inter-sample scenario than in the intra-sample scenario (0.53 ± 0.09 vs. 0.31 ± 0.10; *p* = 0.002). There were also significant differences in accuracy (83 ± 3% vs. 75 ± 7%; *p* = 0.005) and F1 scores (0.64 ± 0.08 vs. 0.46 ± 0.08; *p* = 0.002). The differences were mainly attributed to a significant reduction in sensitivity, from 67 ± 11% in the intra-sample scenario to 43 ± 12% in the inter-sample scenario (*p* = 0.002), whereas we did not find a significant change in specificity (88 ± 4% vs. 87 ± 10%; *p* = 0.64). This performance difference was further highlighted by a significant shift in the composite somatic response distance metric. The distance between predicted and reference (ground truth) response was 0.17 ± 0.03 in the intra-sample scenario, whereas it was 0.25 ± 0.07 in the inter-sample scenario (*p* = 0.005). The difference in precision was not significant (63 ± 10% vs. 55 ± 15%; *p* = 0.16).

To assess whether the VTA information had a relevant contribution to the somatic predictions we performed a control analysis. In this analysis, we trained and tested the predictions using a feature set that excluded the VTA-related features, leaving only *Pw*, *Freq*, *Current*, *Hemisphere*. For the intra-sample scenario, the removal of the VTA-related information led to a significant reduction in Kappa to 0.43 ± 0.06 (*p* = 0.016). Sensitivity dropped from 67 ± 11% to 57 ± 6% (*p* = 0.033), and the F1-score decreased from 0.64 ± 0.08 to 0.56 ± 0.05 (*p* = 0.016). Accuracy dropped from 83 ± 3% to 80 ± 3%, while the composite response distance increased from 0.17 ± 0.03 to 0.20 ± 0.03, with both changes just above the significance threshold (both *p* = 0.064). No significant decreases were observed in specificity (88 ± 4% vs. 87 ± 5%; *p* = 0.35) and precision (63 ± 10% vs. 56 ± 10%; *p* = 0.09). These results indicate that in the intra-sample scenario, the inclusion of spatial VTA information improved somatic predictions. In contrast, the control analysis showed less differences in the inter-sample scenario. Here the reduction in Kappa to 0.30 ± 0.08 was insignificant (*p* = 0.32). However, the reductions in precision (55 ± 16% to 47 ± 8%; *p* = 0.052) and specificity (87 ± 10% to 84 ± 3%; *p* = 0.052) were both close to the significant threshold. No significant changes were observed in the other metrics.

Finally, we compared the prediction performance metrics to the naive baseline predictions, as summarized in Supplementary Figure S3. For both scenarios, Kappa values for the actual predictions were significantly higher than those of the random and constant-baseline models (all *p* < 0.001, with one exception). In the inter-sample somatic predictions, the associated *p*-value (*p* = 0.026) was substantially larger than the *p*-value of the other control comparisons, indicating a more modest level of significance. In line with this finding, the naive predictions revealed a bias caused by a higher proportion (77%) of False instances in the ground truth for the somatic predictions. This was reflected in the accuracy metric of the naive models: Random predictions yielded an accuracy of 73%, while the constant True output (representing the opposite class) resulted in an accuracy of 25%. In light of these observations, the somatic prediction model appears to have marginal predictive power for unseen cases.

## Discussion

In this study, we aimed to predict paresthesias’ occurrence and locations in response to thalamic DBS in individuals with two different clinical symptoms (tremor vs. neuropathic pain), and electrode locations (Vim-nucleus vs. VPL/VPM nuclei, respectively). Using a dataset comprising a large number of stimulation responses obtained empirically from 18 DBS lead implanted in 10 individuals, we explored how stimulation parameters and person specific features influenced perceptual outcomes. Our goal was to evaluate the predictive model’s performance to then in the future assess its real-world relevance for optimizing DBS therapy management and potential applications in computer-brain interfacing.

Our findings reveal several important insights: Firstly, the predictions demonstrated substantial agreement with ground truth for the occurrence of paresthesias, indicating the feasibility of using computational approaches to anticipate sensory outcomes in DBS. Secondly, the importance of incorporating individual-specific information from VTA simulations in predicting the somatic regions of DBS-induced paresthesias highlights the value of image guided DBS programming, underscoring the need to integrate such data in DBS programming and optimization workflow.

The distinction between intra-sample and inter-sample scenarios offers valuable perspective on the real-world application and generalizability of our results. We achieved comparable prediction performance for both previously encountered and unseen cases for the presence of paresthesias. However, the performance of predicting the somatic location of paresthesias decreased for unseen cases. Thus, the model appears to have captured individual characteristics better than generalizable patterns across different individuals. The control analyses with naive models revealed that the model’s somatic predictions for entirely unseen cases were only slightly better than random guessing, indicating that, at this point, the clinical relevance of these predictions may be limited. Somatic predictions seem to require individual-specific prior data, such as stimulation responses collected during prior programming sessions or even during surgery.

We extend earlier research that demonstrated the potential of machine learning models to predict motor response after deep brain stimulation in Parkinson’s disease (36) and Essential Tremor (37). Furthermore, a recent study on people with isolated dystonia demonstrated that machine-learning based DBS programming resulted in greater symptom reduction compared to clinical programming (38). To our knowledge, no prior studies have explored the use of machine-learning approaches for predicting the occurrence and location of paresthesias during DBS. Our findings support the role of sensory afferent fiber activation in mediating paresthesias during DBS (21, 22). However, we build further upon this understanding by demonstrating the predictive utility of computational models incorporating stimulation parameters and VTA metrics. This represents a significant advancement in the field by providing clinicians with a potential tool to anticipate and mitigate sensory side effects associated with DBS therapy.

Our findings could have implications for future clinical practice. Paresthesias can serve as an invaluable real-time feedback signal about the functional effects of DBS. Paresthesias can be a marker of efficacy in spinal cord stimulation (SCS) therapy, correlating with pain relief (39–41). Their role in DBS is less clear, but some studies suggest a similar relationship (42, 43). Furthermore, the location of the paresthesias can offer important information to determine the optimal stimulation site. Specifically, face or finger paresthesias suggests optimal placement within the Vim nucleus for tremor suppression. Conversely, deviations like intra-oral or leg paresthesias could indicate a need for stimulation adjustment, or electrode repositioning (21). Our paradigm can help clinicians predict paresthesias caused by DBS, allowing tailoring of treatment per individual. This has the potential to enhance therapy outcomes, reduce treatment-related morbidity, and improve overall satisfaction with DBS therapy. Moreover, our research supports the integration of computational modeling approaches into clinical practice to augment decision-making and improve patient care in neurostimulation therapies (44).

Our findings extend beyond clinical DBS and are relevant to the field of CBI, particularly in applications requiring precise sensory induction (17, 18). Our prediction paradigm could be leveraged in CBI systems that require modulation of neural activity to evoke specific sensations or perceptions. For instance, by adjusting stimulation parameters and targeting brain regions identified through paresthesia mapping, researchers could design CBI devices capable of generating controlled tactile sensations or proprioceptive feedback. These signals could be used to convey information from the external world, such as artificial touch perceived in a prosthetic limb or a phantom limb (17, 45, 46). Moreover, there is a rich body of work on sensory substitution showing that artificial sensory input can be used to restore, replace, or enhance sensory function (47–51). We have recently demonstrated that paresthesias evoked by spinal cord stimulation can be used to effectively convey a diverse range of information, from rhythmic cues to an artificial sense of balance (18). These advancements could have transformative implications for various CBI domains, including neuroprosthetics, virtual reality, and sensory augmentation technologies. By enabling enhanced sensory experiences and functional restoration for individuals with sensory impairments, our study paves the way for unlocking the full potential of DBS-based CBI, combining valuable insights from clinical therapy, paresthesia mapping, and sensory substitution.

Our study has several limitations. The study’s sample lacks a broad representation of individual characteristics and response variability. On the other hand, the sample’s heterogeneity may introduce confounding factors that could affect the generalizability of the findings. While the study showed promising results for this specific sample of individuals and experimental setting, it’s unclear how well these findings would translate to everyday clinical practice or different subpopulations. Factors such as variations in DBS electrode type and location, stimulation protocols, demographics, and medical condition could impact the model’s performance in real-world applications. Stimulation parameters and anatomical VTA information only partially account for the variation in responses to DBS (1, 52, 53). Unaccounted variables, such as medication or comorbidities, may have influenced the perception of paresthesias. However, we believe these factors are unlikely to introduce significant confounds given our primary objective: predicting stimulation effects using energy-related parameters and anatomical locations. Furthermore, with respect to a potential CBI approach our data does not cover long term paresthesia effects, since it was not the purpose of the paresthesia screening done here. Lastly, while this study provides insights into the predictive modeling of DBS-induced sensations, practical challenges related to model implementation and integration into clinical practice remain.

Looking ahead, several avenues for future research emerge. For example, further refinement and validation of predictive models incorporating larger cohorts, additional features, and biomarkers could enhance the accuracy and clinical utility of paresthesia prediction in DBS therapy. Also, investigations into the broader applicability of predictive modeling approaches across different DBS targets, outcomes, and populations would be valuable for advancing the field of neuromodulation.

## Conclusion

We have demonstrated the feasibility of using machine learning to predict paresthesias in response to thalamic DBS. This predictive modeling provides clinicians with a powerful tool to optimize DBS programming, allowing them to either avoid or intentionally evoke paresthesias. Additionally, paresthesia mapping could enhance computer-brain interfaces, enabling precise sensory induction and offering transformative applications in neuroprosthetics, virtual reality, and sensory augmentation technologies. Our findings are intended to serve as a stepping stone for future, more extensive studies, such as those with larger cohorts. These advancements may pave the way for neuromodulation interventions that are better tailored to individual needs.

## Data Availability

The raw data supporting the conclusions of this article will be made available by the authors, without undue reservation.
