# Supplementary material for: Predictive modeling of sensory responses in deep brain stimulation
Source: Front Neurol. 2024 Oct 1;15:1467307. doi: 10.3389/fneur.2024.1467307 (PMC11473379; doi:10.3389/fneur.2024.1467307)
Supplement: Supplementary file 1 [file Data_Sheet_1.PDF]

Supplementary Materials

SUPPLEMENTARY FIGURES

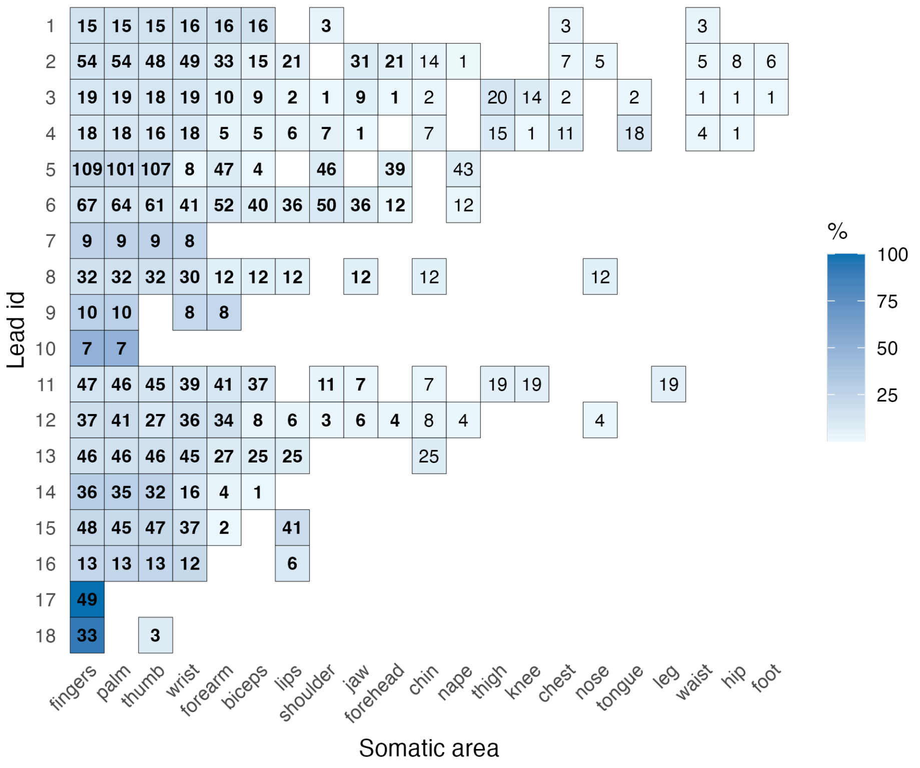

Figure S1. The total number of paresthesias recorded is shown for each somatic area and each DBS lead. These data were used for training and testing the prediction models. The numbers in bold represent trials that were used as positive instances in the somatic area training and predictions. The numbers in regular font represent trials used only as negative instances, meaning they were used to balance out the trials where the respective somatic sensation did not occur (e.g., false instances for the model predicting paresthesias in the fingers).

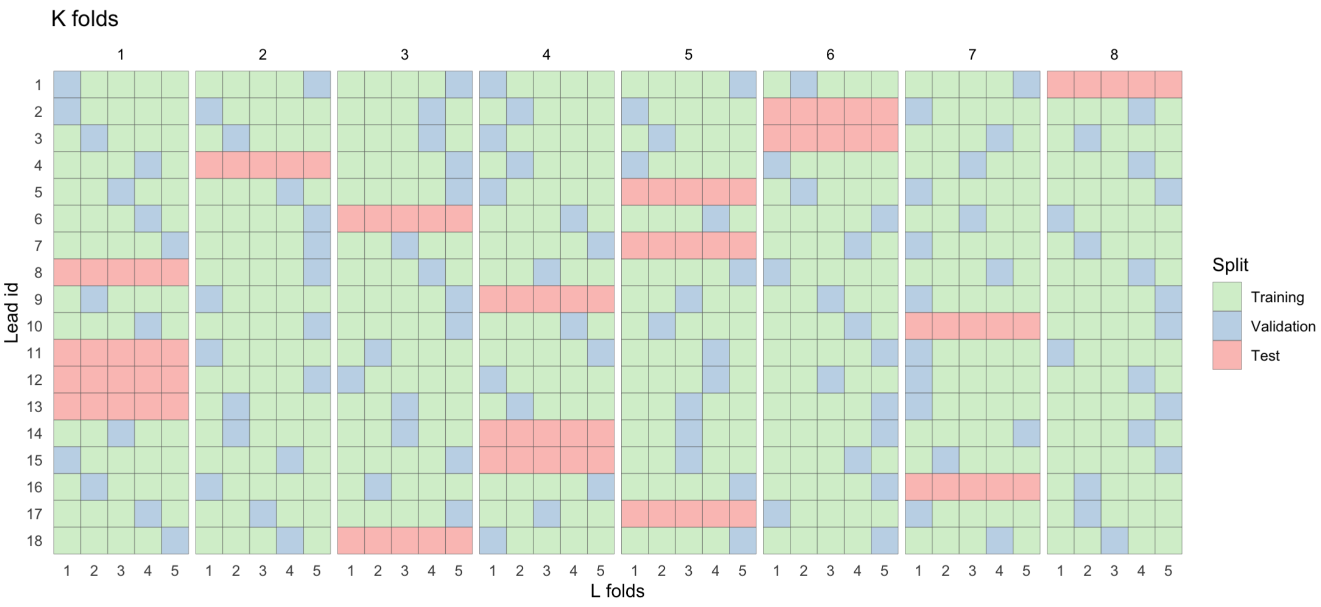

Figure S2. Visualization of K-fold and L-fold splits for training, validation, and test sets across different leads. Each tile represents the split allocation of a specific lead (y-axis) within a particular L-fold (x-axis) under different K-folds (facets). The color coding indicates whether the lead was used for training, validation, or testing during the model fitting process. In this scenario, we had homogeneous K and L fold sizes, but this may not always since the fold creation is partly random. The fold creation resulted in an average split ratio of 87.5% (training) vs. 12.5% (testing) for the K-folds, and 80% (training) vs. 20% (validation) within the L folds.

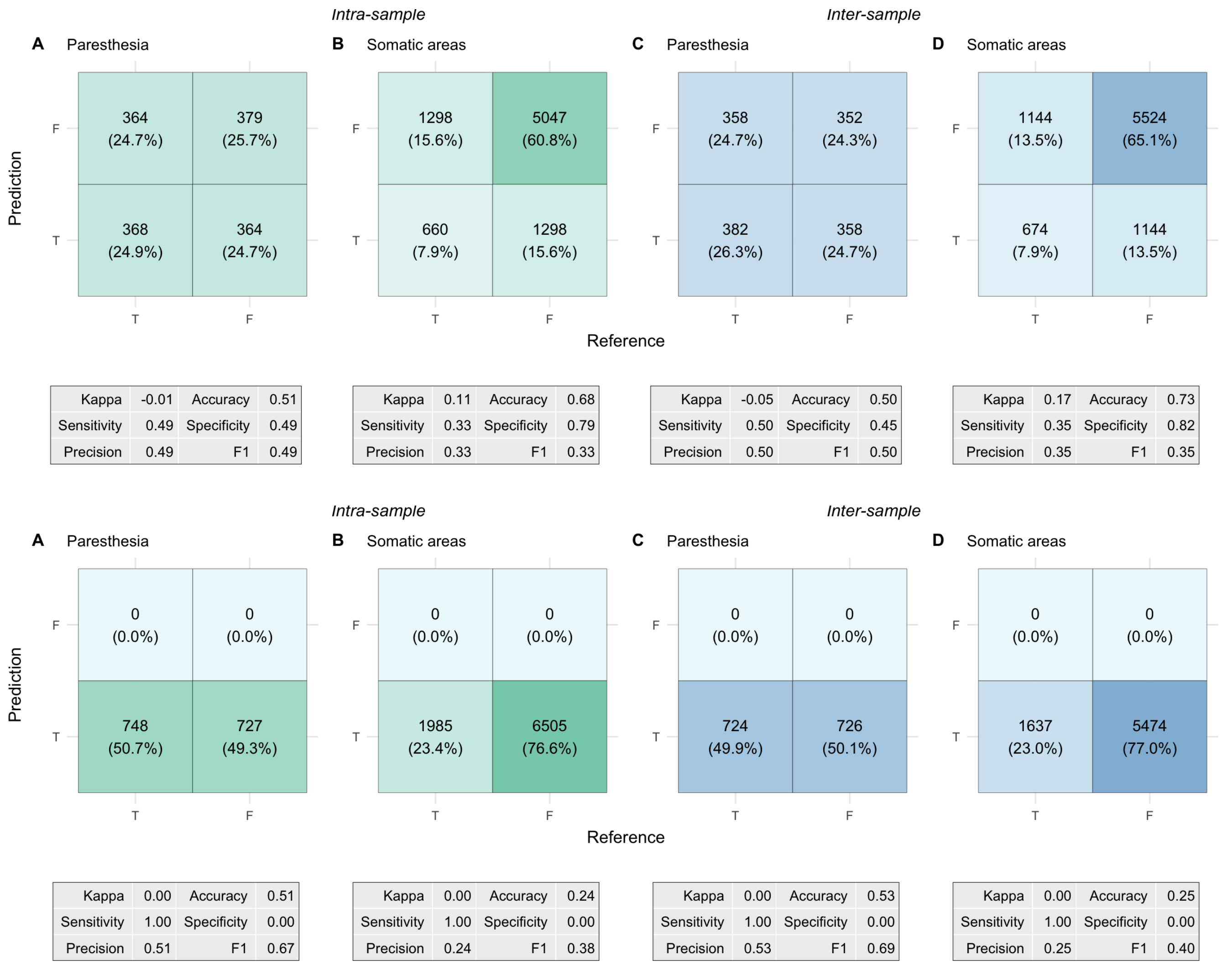

Figure S3. Confusion matrices and performance metrics from control analyses where nested cross-validation was performed using naive baseline models that did not incorporate or use stimulation-related features. Each cell shows the total count of true/false positives/negatives, with the corresponding percentage in parentheses. Percentages represent the proportion of total predictions classified as True (paresthesia occurring) or False (paresthesia not occurring), with model predictions on the vertical axis and the reference (ground truth) on the horizontal axis. The panels depict: *A)* Intra-sample paresthesia predictions, *B)* Intra-sample somatic predictions, *C)* Inter-sample paresthesia predictions, and *D)* Inter-sample somatic predictions. The tables beneath each matrix show the corresponding performance metrics. **Top row:** Results from naive models that made predictions by randomly permuting the reference (ground truth) response data. **Bottom row:** Results from naive models that always predicted True for both paresthesia occurrence and somatic area.
